# Diversity and Conservation Gap Analysis of the Solanaceae of Southern South America

**DOI:** 10.3389/fpls.2022.854372

**Published:** 2022-05-17

**Authors:** Andrés Moreira-Muñoz, María Virginia Palchetti, Vanezza Morales-Fierro, Valeria Soledad Duval, Rudy Allesch-Villalobos, Carlos E. González-Orozco

**Affiliations:** ^1^Instituto de Geografía, Pontificia Universidad Católica de Valparaíso, Valparaíso, Chile; ^2^Instituto Multidisciplinario de Biología Vegetal - IMBIV, CONICET, Universidad Nacional de Córdoba, Córdoba, Argentina; ^3^Departamento de Ciencias Farmacéuticas, Facultad de Ciencias Químicas, Universidad Nacional de Córdoba, Córdoba, Argentina; ^4^Museo Nacional de Historia Natural, Interior Parque Quinta Normal S/N, Santiago, Chile; ^5^Departamento de Geografía y Turismo, Universidad Nacional del Sur, Bahía Blanca, Argentina; ^6^Corporación Colombiana de Investigación Agropecuaria- Agrosavia, Centro de Investigación La Libertad, Meta, Colombia

**Keywords:** micro-hotspots, conservation biogeography, plant blindness, protected areas, biodiverse

## Abstract

There is a need to make substantial advances in the taxonomic, systematic, and distribution knowledge of plants, and find better ways of transmission of this information to society to surpass the general pattern described as “plant blindness.” The diversity of the plant family Solanaceae reaches its peak in South America; however, many of its species are threatened due to the expansion of the human footprint. Here, we examine the diversity patterns of the family in southern South America (Argentina and Chile) by means of species richness (SR), weighted endemism (WE), and corrected weighted endemism (CWE). We also evaluated conservation gaps in relation to protected areas and the human footprint as a proxy for potential impacts on this biodiversity. Results show two richness centers in NW and NE Argentina, with a high degree of overlap with protected areas, which, on the other side, show a relative high index of human footprint. Comparatively, coastal Atacama (Chile) shows lower richness values, but outstanding CWE and WE values. The coast of Atacama harbors high values due the presence of species of the genus *Nolana* with restricted distributions. Protected areas in this tight coastal strip are sparse, and the human footprint is also relatively high. The degree of protection based on these parameters is then unbalanced, highlighting the need for a geographically explicit strategy for the conservation of the family at subcontinental scale. In doing so, it is likely that other representatives of these unique centers of richness and endemism will benefit.

## Introduction

Plant conservation is limited by our knowledge of the diversity, distribution, and abundance of plant species (Gillson et al., 2020). This information is increasing but cannot keep pace with the threats plants suffer, leading to accelerated anthropogenic-caused extinctions and genetic erosion (Knapp, 2019; Wandersee and Schussler, 2001).

South America is one region where different approaches (e.g., taxonomy, phylogeny, biogeography, ethnobotany) are leading to the discovery of new species. Unfortunately, this diversity is dwindling across the continent (Ramirez-Villegas et al., 2012). Rapid land use changes, wildfires, and, in general, human footprint expansion (Zalles et al., 2021) are putting species and ecosystems increasingly under threat. Regional climate change amplifies these threats across biodiversity hotspots (Fuentes-Castillo et al., 2020).

Of special interest for plant conservation at a continental and subcontinental scale are several angiosperm groups that exhibit early diversification in South America, including the Bignoniaceae, Verbenaceae, Asteraceae, and Solanaceae (Olmstead, 2013; Dupin et al., 2017; Deanna et al., 2020). Obtaining a comprehensive understanding of the diversity and distribution of these taxa in South America is a challenge, but this effort is paramount to guide future conservation efforts.

The family Solanaceae encompasses approximately 2,800 species globally (98 genera). It is among the 10 families with the greatest diversity in countries considered to be megadiverse, such as Ecuador and Bolivia (Ulloa Ulloa et al., 2017). In addition, many solanaceous species are important food resources (Samuels, 2015).

Representatives of the Solanaceae family are distributed in the Americas from Alaska to Patagonia, from the sea level to the heights of the Andes (e.g., *Solanum acaule, Jaborosa squarrosa*, and *Lycium humile* (Barboza, 2013; Palchetti et al., 2021). Therefore, the family has inspired important biogeographic studies across the continent (Hijmans and Spooner, 2001; Anguiano-Constante et al., 2018).

**FIGURE 1:**
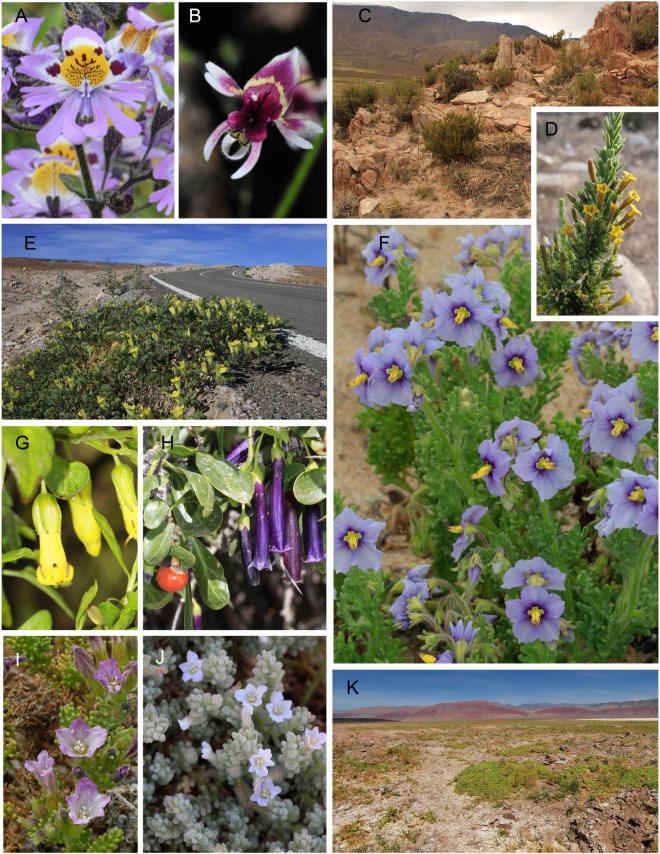
Floral morphology and habitat diversity of southern Solanaceae. **(A)** *Schizanthus porrigens* and **(B)** *Schizanthus parvula* from Central Chile; **(C)** *Fabiana denudata* in Catamarca, Argentina; **(D)** *Fabiana ramulosa* in Chilean Altiplano; **(E)** *Exodeconus flavus* in Tarapacá Precordillera, north Chile; **(F)** *Solanum trinominum* in Chilean coastal sand dunes; **(G)** *Salpichroa glandulosa* and **(H)** *Dunalia spinosa* in the heights of Parinacota, Chile; **(I,J)** *Nolana mollis* and *N. villosa* on the lomas formation, Chile; **(K)** *Lycium humile* on salt-rich environments of the Altiplano highlands. Photographs by M. Virginia Palchetti, Rocío Deanna, and Andrés Moreira-Muñoz.

The southern end of the continent is where the family reaches the greatest levels of diversity as the fifth largest family of the flora of the Southern Cone of South America after the Asteraceae, Poaceae, Fabaceae, and Orchidaceae (Zuloaga et al., 2019) (Figure 1).

The type genus of the family is *Solanum*, a genus of nearly cosmopolitan distribution, published by Linnaeus (1753) in Species Plantarum. It is the second most diverse genus of the vascular plants in the Southern Cone (216 spp.) after *Senecio* (Asteraceae, 415 spp.) (Zuloaga et al., 2019).

Solanaceae is the fourth family in species richness in Argentina (Palchetti et al., 2020) as it is in Chile (Moreira-Muñoz, 2011). With 52% of endemic species, Chile stands out as the most relative endemism-rich country for the Solanaceae, followed by the megadiverse Peru and Brazil (47%) (Palchetti et al., 2020). More diverse genera in Argentina are *Solanum* and *Lycium*, while, in Chile, the highest diversity is represented by *Solanum* and *Nolana*. In Argentina, ecoregions with highest diversity are Chaco, Andes, and Pampa, and highest endemism occurs in Chaco, Andes, Yungas, and Monte ecoregions (Oyarzabal et al., 2018; Del Valle Elías and Aagesen, 2019; Palchetti et al., 2020; Arana et al., 2021). In Chile, the most outstanding ecoregion is the Desert Scrub, as defined by Luebert and Pliscoff (2017). The southern Andes has played a central role in the early diversification of the Solanaceae. The history of elevation change in the Andes occurred concurrently with plant evolution and influenced it, the mountains acting as a corridor, a barrier or providing a geodiversity framework for species diversification (Luebert and Weigend, 2014; Moreira-Muñoz et al., 2020).

Recent advances in the knowledge of the taxonomy of this family have challenged us to update the overall understanding of diversity and conservation priorities. Ca. 30 species are considered as threatened in Argentina (Palchetti et al., 2020) and 14 species in Chile, but most species have not been assessed yet.^1^

Our main goal in this study was to map and analyze the diversity of the Solanaceae in southern South America (Argentina and Chile), overlaying regional richness and endemism with protected areas to identify conservation gaps. Additionally, the human footprint in the existing protected areas is evaluated as a proxy for the degree of effective protection of the family. In this way, the areas of geographic concentration (richness micro-hotspots and centers of endemism) of the family can be identified, which can guide future floristic prospecting and identify areas under threat from land uses incompatible with conservation.

## Methods

The distribution and richness analysis for the family was carried out through a compilation of a database, including different sources of information. Data for Argentina come mostly from the Documenta Florae Australis (2021), while the data for Chile collate specimen information from national (CONC and SGO) and international herbaria. The latter are available through the Global Biodiversity Information Facility (GBIF) platform. Specific status based on recent studies and reports published after April 2019 (not included in Palchetti et al., 2020) has been considered. This included studies, such as the revision of the genus *Schizanthus* (Morales-Fierro et al., 2020; Lavandero et al., 2021); updates in *Nolana* (Hepp and Dillon, 2018); the Morelloid clade of *Solanum* in Argentina (Knapp et al., 2020); cryptic species recently reported (Moreira-Muñoz and Muñoz-Schick, 2020); and new *Petunia* and *Nicotiana* species (Greppi et al., 2019; Santilli et al., 2021). After a first cleaning, data from *ex situ* living collections that introduced species, hybrid taxa, and records with doubtful or incomplete identification at the species level were excluded. Despite the fact that most of the records had georeferenced data in their originally source, errors were detected and corrected (19% of the total records). For this purpose, the Geonames^2^ and Mapcarta^3^ sites were used. Species nomenclature was based on the following sources: Flora del Cono Sur,^4^ POWO,^5^ and Solanaceae Source.^6^ After eliminating duplicate coordinates for each species, the final database consists of 15,510 records, which include 35 genera and 423 species (Supplementary Material).

Diversity maps were carried out by means of the Biodiverse 3.1 software. (Laffan et al., 2010).^7^ We used grid cells of 1 degree (latitude and longitude) (the most suited resolution at subcontinental scale), and three diversity indices were computed from the grid-cell data. The species richness (SR) of a cell is defined as the total number of species within that grid cell. Weighted endemism (WE) is the sum, over all species present in the window for that grid cell, of the number of grid cells in the window with that species divided by the range of that species. The range is defined as the total number of all grid cells in which that species is present. Crisp et al. (2001) defined corrected weighted endemism CWE as the weighted endemism (WE) divided by the total number of species in that window. This last division adjusts the index for the effect of SR. To assess the confidence in the identified centers of endemism, we conducted a randomization test (Laffan and Crisp, 2003). The test generated 999 random iterations, each of which preserves the observed SR of each geographically located cell, and the total number of cells, or ranges, for each species. CWE was then calculated for each random iteration, and the original ranked against the randomizations. Cells with CWE randomization ranks in the top 5% are significantly different from random at a threshold of alfa = 0.05 (González-Orozco et al., 2011) (Supplementary Material).

Gap analysis was carried out by means of the superposition of the richness units with available information on protected areas. Argentina-protected units were a downloaded official site.^8^ Chilean units are available on the SNIT Geoportal.^9^ Additionally, as a proxy for the state of protection of species within protected areas, we overlayed them with the “human footprint” index, as developed by Sanderson et al. (2002) by means of ArcGis 10.3 (ESRI, 2015). The human footprint is a quantitative analysis and representation of human influence across the planetary surface based upon four types of data: population density, land transformation, accessibility, and electrical power infrastructure. Human impact is represented on a scale of 0 (minimum) to 100 (maximum) on a resolution of one square kilometer (Sanderson et al., 2002). A score of 1 indicates the least human influence. The shape file for South America was obtained from^10^ and overlapped with the protected areas in Chile and Argentina. We calculated the mean of the human footprint in the set of pixels, encompassing a protected area (Table 1 and Supplementary Material).

**TABLE 1:** Protected areas in Argentina and Chile with the higher number of Solanaceae species.

Argentina	No species	Footprint index
Reserva de Biosfera de las Yungas	77	18.3
Patrimonio Cultural de la Humanidad Quebrada de Humahuaca	54	17.5
Reserva Natural Provincial del Iberá	50	11.8
Sitio Ramsar Humedales Chaco	43	28.6
Parque Provincial Cumbres Calchaquíes	40	19.0

**Chile**	**No species**	**Footprint index**

Parque Nacional Pan de Azúcar	22	17.2
Monumento Natural Paposo Norte	19	26.7
Parque Nacional Fray Jorge	17	36.2
Parque Nacional Morro Moreno	12	26.8
Parque Nacional Llanos de Challe	9	19.7

## Results

### Taxonomic Diversity

The number of native Solanaceae species in Argentina and Chile is 430 and belongs to 35 genera. Both countries share 18 genera and 55 species, of which 27 are endemic to Argentina and Chile. Argentina has 315 native species, distributed in 32 genera, of which 80 are endemic species (25%). Chile has 170 native species, distributed in 21 genera, of which 89 are endemic species (52%). Supplementary Material considering Argentina and Chile, a total of 8 genera are endemic (i.e., *Benthamiella*, *Combera*, *Reyesia*, *Salpiglossis*, and *Schizanthus* shared between countries; the monotypic genus *Panthacantha* only grows in Argentina, and the monotypic genera *Latua* and *Vestia* in Chile). The most speciose genus in Chile is *Nolana*, with 49 native species, while in Argentina is *Solanum*, with 126 native species.

### Spatial Patterns of Biodiversity

**FIGURE 2:**
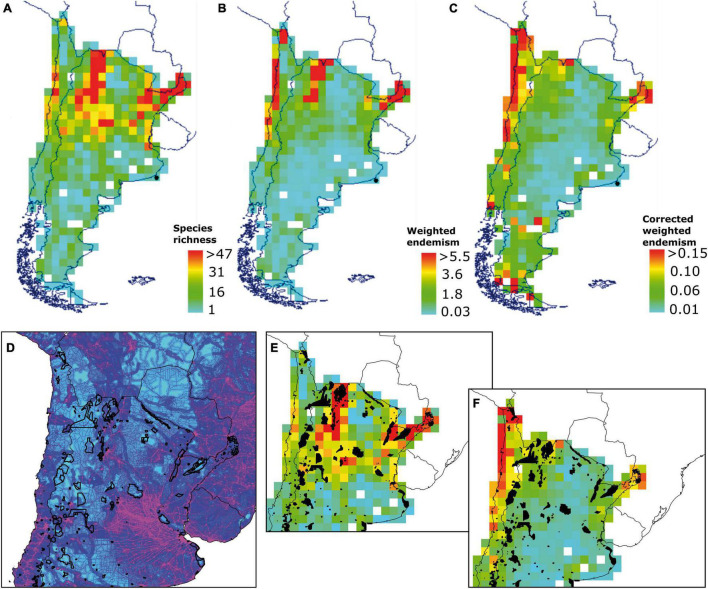
Diversity of Solanaceae species in southern South America, mapped using Biodiverse 3.1 on 1 × 1 degree matrix: **(A)** Species richness; **(B)** Weighted endemism; **(C)** Corrected weighted endemism; **(D)** A detailed area showing a footprint index and protected areas; **(E)** Protected areas overlapped with species richness; **(F)** Protected areas overlapped with corrected weighted endemism. Detailed maps and tables are available as (Supplementary Material).

According to Biodiverse 3.1 outputs, the primary centers of SR are in the northeast and northwestern regions of Argentina and coastal areas in the north of Chile (Figure 2). The SR scores ranged between 1 and 111 species, but SR maximum value of 47 at a threshold of 5–95% was found in a single-grid cell. We identified three main hotspots of WE: east and west northern corner of Argentina and the coastal areas in the north of Chile. WE scores ranged between 0.007 and 11.71, but a WE maximum value of 5.5 (5.5% of species are endemic to that grid cell) at a threshold of 5–95% was found in a single grid cell (Figure 2). Once species richness is being corrected, two of the main WE centers remained in the same location, and the one in the northwestern corner of Argentina tended to disappear. However, new areas of high CWE appeared in the south of Chile and Argentina as well as a few scattered grid cells in the central regions. Interestingly, the northern hotspot of endemism in Chile increases in size under the CWE. These changes are likely because of richness biases on specific grid cells. CWE scores ranged between 0 and 0.50, but a CWE maximum value of 0.15 (after a correction of richness, 15% of species are endemic to that grid cell) at a threshold of 5–95% was found in a single-grid cell. To test the validity of spatial CWE patterns, the randomization results show that all major identified centers of endemism were significantly different from random at a threshold of alfa = 0.05 (Figure 2).

In synthesis, NW and NE Argentina and the northern Atacama coast can be considered as richness/endemism centers, or micro-hotspots of biodiversity, at the margins of globally recognized biodiversity hotspots (Supplementary Material).

### Conservation

Gap analysis shows that, from 331 protected areas in Argentina, 129 have a degree of spatial coincidence with the distribution of Solanaceae. In Chile, 38 from 102 protected units superpose with cells with the distribution of Solanaceae. Protected areas with highest number of species in Chile and Argentina are those summarized in Table 1. Protected areas in the northwest of Argentina show a spatial match with richness centers and, to some lower degree, in NE Argentina (Figure 2E). The presence of protected areas in Chile is sparse at the coast of Atacama, where the highest endemism appears. The human footprint is higher in Central Chile and Central/northern Argentina, around metropolitan central areas (Supplementary Material). The mean value of the human print index in each protected area (PA) varies between 1.1 and 87 in Argentina (20.1 total mean). The index varies between 1.8 and 71 in the case of PA in Chile (19.7 total mean). PA areas encompassing high numbers of Solanaceae and a relatively high index are Sitio Ramsar Humedales Chaco in Argentina and Parque Nacional Fray Jorge in Chile (Table 1).

## Discussion

One of the greatest current challenges in conservation biogeography is identifying areas of high species richness and endemism, both to establish conservation priorities and to better understand the evolution of plant diversity. This is especially relevant in southern South America, a territory recognized as especially important in the evolution of diverse families, such as Solanaceae, Bignoniaceae, Verbenaceae, Asteraceae, Orchidaceae (Olmstead, 2013; Ulloa Ulloa et al., 2017). In southern South America, the wide geographic distribution of the family Solanaceae and its taxonomic richness is partly explained by a long evolutionary history since the early Eocene (Dupin et al., 2017; Deanna et al., 2020).

Diversity indexes applied to this wide study area show different regions as outstanding for Solanaceae species richness (SR) and endemism, respectively. Weighted endemism (WE) and corrected weighted endemism (CWE) are parameters that have shown great utility for analysis of restricted distributions (Sosa and de Nova, 2012; Rodríguez et al., 2018; Ruiz-Sánchez et al., 2020). SR shows two main areas in NW and NE Argentina. Richness areas show an important degree of protection mainly by “Reserva de Biosfera de las Yungas” and “Patrimonio Cultural de la Humanidad Quebrada de Humahuaca” in the NW, and “Reserva Natural Provincial del Iberá” and “Sitio Ramsar Humedales Chaco” in the NE. Both regions have been largely recognized as important sources of biodiversity and medicinal plants (Hilgert and Gil, 2006; Bernacki et al., 2015; Campanello et al., 2019). Especially Yungas has been recognized as an outstanding ecoregion for the conservation of different biotic groups (Grosso and Quintana, 2009; Arana et al., 2016; Torres and González-Reyes, 2017). When weighted endemism (WE) and corrected weighted endemism (CWE) are applied, another region clearly appears as outstanding at the regional scale: the coast of Atacama, mainly due the diversity and restricted distribution of species in genus *Nolana*. This genus is a main component of Lomas vegetation, a plant formation found along the coast from Peru to northern Chile, characterized by high endemism and richness maintained by the coastal fog reaching the coastal cliffs at an altitude around 1,000 m asl (Muñoz-Schick et al., 2001). The Solanaceae component of this remarkable environment has been emphasized by Dillon (2005, 2016), and other components of this unique biota have been recently highlighted (Moat et al., 2021; Pizarro-Araya et al., 2021).

The three outstanding centers of richness and endemism (micro-hotspots) of southern Solanaceae species are part of three different biodiversity hotspots at a continental scale: the northwest of Argentina is at the margin of the Tropical Andes hotspot; the northeast is at the margin of the Atlantic Forest hotspot, and the coastal Atacama is adjacent to the northern end of the Central Chile hotspot (Supplementary Material). Hotspots are defined as large areas of high species richness subjected to intense threats and landscape modification, as it gets clear by the expansion of the human footprint at a continental scale (Zalles et al., 2021). At least in Chile, protected areas have huge deficits in effective protection (Petit et al., 2018), and, certainly, much more efforts shall be done for effective protection at the landscape level, including target families, such as the Solanaceae. Results presented here remark the need for a geographically explicit strategy for the conservation of the family Solanaceae at subcontinental scale. In doing so, it is likely that other representatives of these centers of richness and endemism will benefit, promoting conservation and restoration at the landscape scale (Ianni and Geneletti, 2010; Malizia et al., 2012), and hopefully contributing also to reduce the generalized “plant blindness.”

## Author Contributions

AM-M and MP conceived the study and wrote the manuscript. VM-F compiled, cleaned, and updated the data base. VM-F and RA-V ran GIS analysis by means of ArcGis. VSD discussed the implications of biodiversity values in relation to protected areas. CG-O ran Biodiverse 3.1 analysis and analyzed results. All authors edited the manuscript and approved the submitted version.

## Conflict of Interest

The authors declare that the research was conducted in the absence of any commercial or financial relationships that could be construed as a potential conflict of interest.

## Publisher’s Note

All claims expressed in this article are solely those of the authors and do not necessarily represent those of their affiliated organizations, or those of the publisher, the editors and the reviewers. Any product that may be evaluated in this article, or claim that may be made by its manufacturer, is not guaranteed or endorsed by the publisher.

## Footnotes

1
https://clasificacionespecies.mma.gob.cl/

2
https://www.geonames.org/

3
https://mapcarta.com/

4
http://www.darwin.edu.ar

5
http://www.plantsoftheworldonline.org

6
http://solanaceaesource.org/

7
http://shawnlaffan.github.io/biodiverse/

8
https://www.argentina.gob.ar/ambiente/areas-protegidas

9
http://www.geoportal.cl/geoportal/catalog/main/home.page

10
https://earthdata.nasa.gov/learn/sensing-our-planet/the-human-footprint
