# Supplementary figures and images for: Diversity and Conservation Gap Analysis of the Solanaceae of Southern South America
Source: Front Plant Sci. 2022 May 17;13:854372. doi: 10.3389/fpls.2022.854372 (PMC9152431; doi:10.3389/fpls.2022.854372)

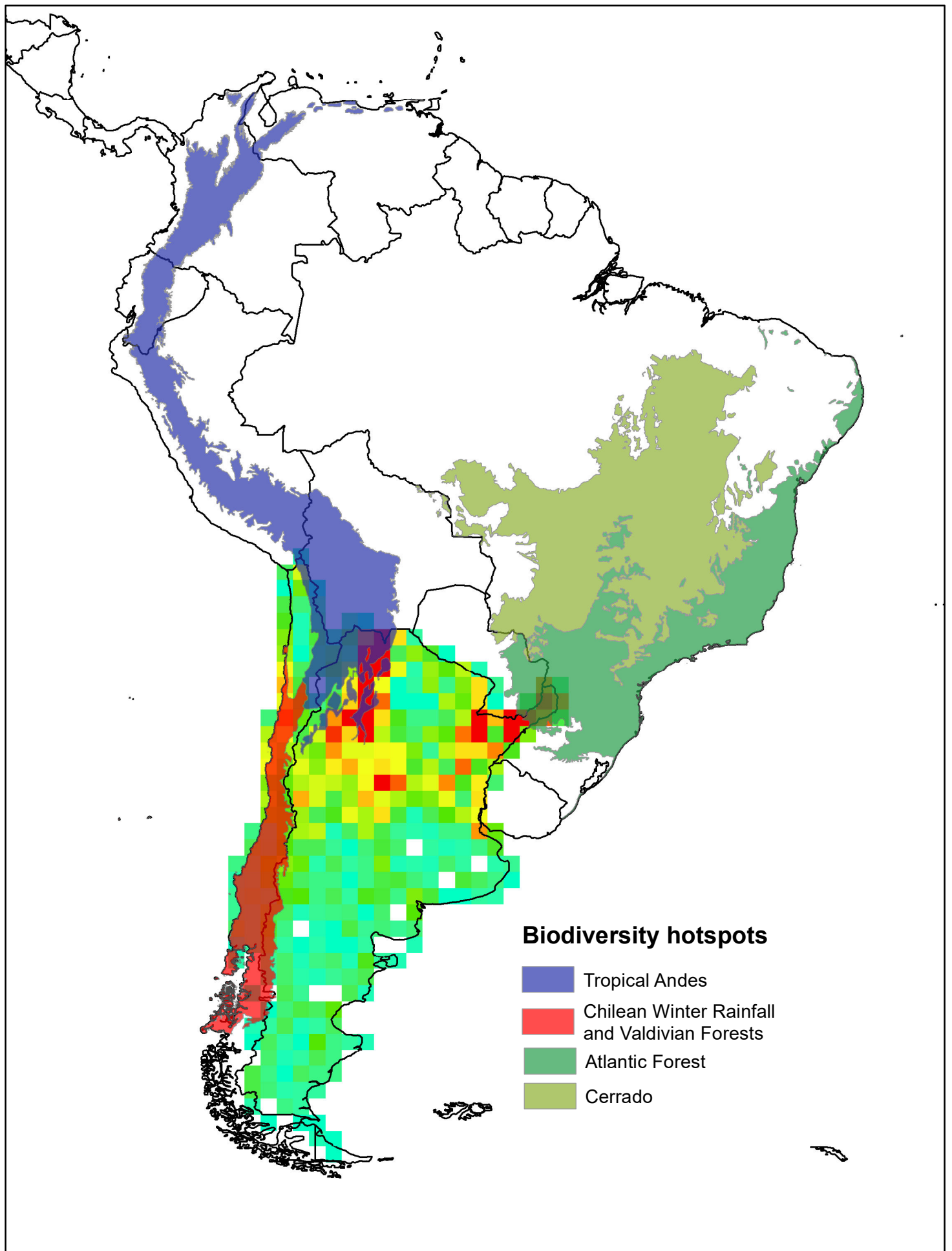

Supplement: Supplementary file 5 [file Data_Sheet_2.PDF]

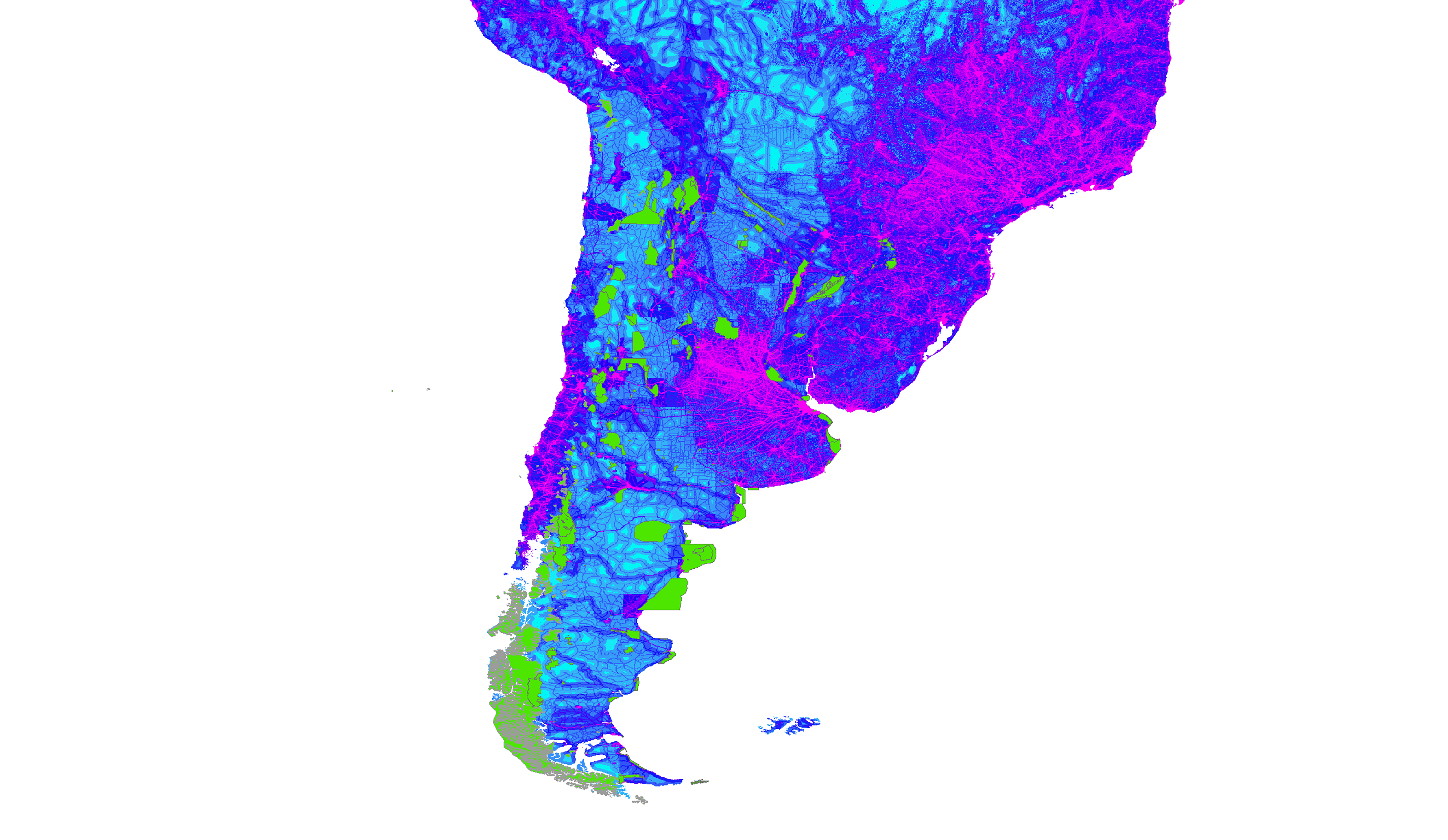

Supplement: Supplementary file 6 [file Image_1.PNG]
